# Bioinformatics and System Biology Approach to Reveal the Interaction Network and the Therapeutic Implications for Non-Small Cell Lung Cancer Patients With COVID-19

**DOI:** 10.3389/fphar.2022.857730

**Published:** 2022-06-02

**Authors:** Zhenjie Zhuang, Xiaoying Zhong, Qianying Chen, Huiqi Chen, Zhanhua Liu

**Affiliations:** ^1^ Guangzhou University of Chinese Medicine, Guangzhou, China; ^2^ Department of Oncology, The First Affiliated Hospital of Guangzhou University of Chinese Medicine, Guangzhou, China

**Keywords:** COVID-19, non-small-cell lung cancer, interaction network, bioinformatic analysis, systemic biological analysis

## Abstract

**Background:** Severe acute respiratory syndrome coronavirus 2 (SARS-CoV-2), the leading cause of coronavirus disease-2019 (COVID-19), is an emerging global health crisis. Lung cancer patients are at a higher risk of COVID-19 infection. With the increasing number of non-small-cell lung cancer (NSCLC) patients with COVID-19, there is an urgent need of efficacious drugs for the treatment of COVID-19/NSCLC.

**Methods:** Based on a comprehensive bioinformatic and systemic biological analysis, this study investigated COVID-19/NSCLC interactional hub genes, detected common pathways and molecular biomarkers, and predicted potential agents for COVID-19 and NSCLC.

**Results:** A total of 122 COVID-19/NSCLC interactional genes and 21 interactional hub genes were identified. The enrichment analysis indicated that COVID-19 and NSCLC shared common signaling pathways, including cell cycle, viral carcinogenesis, and p53 signaling pathway. In total, 10 important transcription factors (TFs) and 44 microRNAs (miRNAs) participated in regulations of 21 interactional hub genes. In addition, 23 potential candidates were predicted for the treatment of COVID-19 and NSCLC.

**Conclusion:** This study increased our understanding of pathophysiology and screened potential drugs for COVID-19 and NSCLC.

## Introduction

Severe acute respiratory syndrome coronavirus 2 (SARS-CoV-2), responsible for pandemic coronavirus disease-2019 (COVID-19), is a novel beta-coronavirus belonging to the subgenus *Sarbecovirus* (Ciotti et al., 2020; Umakanthan et al., 2020). As an emerging global health crisis, SARS-CoV-2 shares a similar transmission mode with other respiratory viruses, mainly through air droplets and close contact (Shang et al., 2021). Due to the rapidly evolving nature of SARS-CoV-2, as on 21 November 2021, there had been 257,788,585 confirmed cases with mortality calculated at 2% (Worldometer, 2021). Recently, the development of Delta and Omicron variants has further complicated the control of the pandemic (Lopez Bernal et al., 2021; Wang and Powell, 2021). About 80% of COVID-19 patients exhibit mild to moderate clinical manifestations, including fever, dyspnea, dry cough, and acute pneumonia (Liu et al., 2020; Mallah et al., 2021; Shang et al., 2021). The case fatality rate for COVID-19 shows a close connection between age, underlying disease status, and immune state (Olloquequi, 2020). A growing body of evidence shows that cancer patients harbor a higher risk of COVID-19 infection, along with severe events and unfavorable outcomes (Passaro et al., 2021; Sinha and Kundu, 2021). A cohort study of 1,590 COVID-19 cases reported that lung cancer was the most frequent type of cancer [5 (28%) of 18 cancer patients] due to the inherent associated pulmonary fragility (Liang et al., 2020). Moreover, lung cancer patients with smoking-related lung damage, significant cardiovascular or respiratory comorbidities, and older age are more likely to develop COVID-19 severity (Berlin et al., 2020; Liang et al., 2020; Passaro et al., 2020). According to the Global Cancer Statistics in 2020, lung cancer remains to be the leading cause of cancer incidence and mortality, representing 11.4% and 18.0% of all cases, respectively (Sung et al., 2021). Accounting for approximately 85% of lung cancers, non-small-cell lung cancer (NSCLC) is comprised of several histological subtypes such as lung adenocarcinoma, squamous-cell carcinoma, and large-cell carcinoma (Zappa and Mousa, 2016). Despite chemotherapy and targeted therapies being widely applied for the treatment of NSCLC, the 5-year survival rate has remained abysmally low (16%) for the last four decades (Suresh et al., 2019).

Cell proliferation is a vital and fundamental mechanism for growth, development, and regeneration of eukaryotic organisms (Diaz-Moralli et al., 2013). Dysregulation of the cell cycle leads to aberrant cell proliferation, which is found in various malignancies (Williams and Stoeber, 2012). Most NSCLCs have detectable cell cycle abnormalities, and the more defective the cell cycle becomes, the more severe the consequences would be (Sterlacci et al., 2012). Driven by complex interactions between host factors, tumorigenesis creates an ideal tumor microenvironment and promotes tumor formation (Dzobo, 2021). Prior studies have pointed out that the tumor microenvironment not only played an important part in tumor development at primary and metastatic sites but also deteriorated viral infection (Tian et al., 2020; Malkani and Rashid, 2021). In lung cancer patients, the tumor microenvironment supports SARS-CoV-2 proteins by activating cytokine storm- and cellular metabolic variation-related pathways, which further accelerate infection and weaken the immune system. Angiotensin-converting enzyme 2 (ACE2), an entry receptor for SARS-CoV-2, almost ubiquitously present in human organs, but primarily expressed in alveolar epithelial type II cells, secretes surfactant and plays a crucial part in pulmonary gas exchange (Zhou P. et al., 2020; Walls et al., 2020). In lung cancer tissues, significantly upregulated ACE2 caused lung parenchyma to become vulnerable to SARS-CoV-2 attack (Zhang L. et al., 2020; Mason, 2020). By triggering associated cascades, SARS-CoV-2 infections increased inflammatory mediators, which could induce paracrine senescence through prolonging cytokine signaling. Accumulative evidence suggests that cellular senescence damages vascular functions persistently by impairing endothelium and decreasing angiogenesis. Vascular dysfunction strikes the balance between anti- and procoagulant pathways and further increases the risk of abnormal coagulation (thrombosis) (Bochenek et al., 2016; Escher et al., 2020). To conclude, persistent inflammation and cellular senescence are associated with pulmonary parenchyma injury, which could potentially restrict blood flow to the lungs, activate coagulation, induce capillary damage, and eventually contribute to hypoxemia and acute respiratory distress in COVID-19 and NSCLC patients.

**FIGURE 1:**
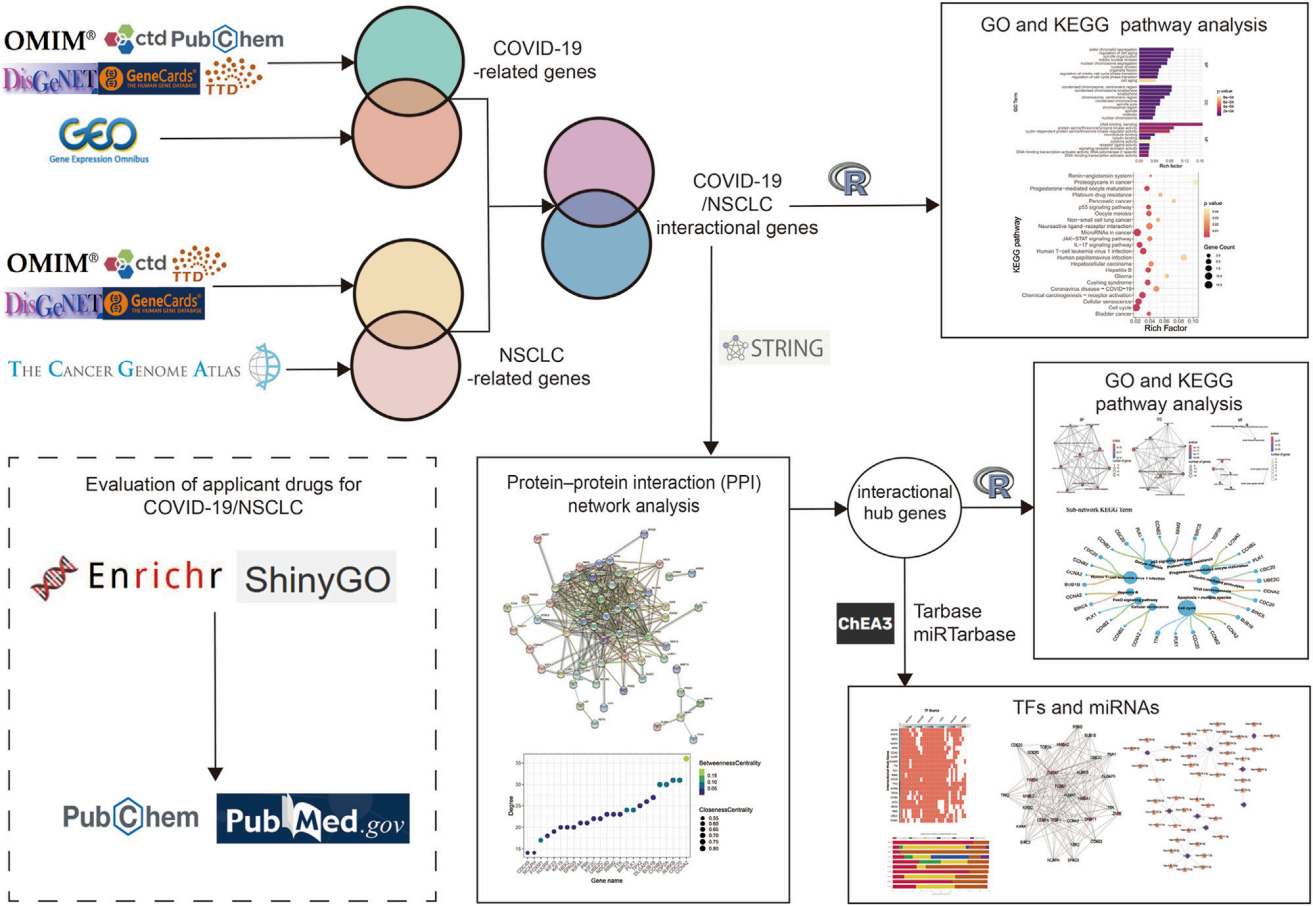
Overview of the workflow of the present study.

In the context of the highly contagious COVID-19 pandemic, the number of NSCLC patients with COVID-19 continues to rise (Worldometer, 2021). Although remdesivir (Eastman et al., 2020; Hung et al., 2020), ribavirin, and hydroxychloroquine (Yao et al., 2020) have been approved for COVID-19, none of them have been suggested to be specific. With the widespread application of high-throughput technologies, a large amount of biological data has been generated. We performed a comprehensive bioinformatic and systemic biological analysis to further understand mechanisms and seek potential efficacious drugs to combat COVID-19/NSCLC (Figure 1).

## Materials and Methods

### Identification of Genetic Interrelations Between COVID-19 and Non-Small-Cell Lung Cancer

To determine genetic interrelations shared by COVID-19 and NSCLC, we searched a variety of databases to collect available data for each disease. We searched six online databases for COVID-19–related genes, including Online Mendelian Inheritance in Man (OMIM, https://www.omim.org/) (Hamosh et al., 2005), Therapeutic Target Database (TTD, http://db.idrblab.net/ttd/) (Wang et al., 2020), PubChem (https://pubchem.ncbi.nlm.nih.gov/) (Kim S. et al., 2019), DisGeNET (https://www.disgenet.org/covid/diseases/summary/) (Piñero et al., 2020), GeneCards (https://www.genecards.org/) (Stelzer et al., 2016), and Comparative Toxicogenomics Database (CTD, http://ctdbase.org/) (Davis et al., 2021). Transcriptomic RNA-sequencing (RNA-seq) datasets of COVID-19 [GSE147507 (Daamen et al., 2021), GSE157103 (Overmyer et al., 2021), and GSE166190 (Vono et al., 2021)] were downloaded from Gene Expression Omnibus (GEO, https://www.ncbi.nlm.nih.gov/geo/) (Clough and Barrett, 2016). Then, we used the R package *Deseq2* (Love et al., 2014) to extract differentially expressed genes (DEGs) at the threshold |log2FoldChange| >1 and adjusted the p-value <0.05. COVID-19–related genes were identified by intersecting related genes from online databases and GEO datasets.

In addition, five online platforms were searched for NSCLC-related genes, including OMIM (Hamosh et al., 2005), CTD (Davis et al., 2021), TTD (Wang et al., 2020), DisGeNET (Piñero et al., 2020), and GeneCards (Stelzer et al., 2016). RNA-seq datasets of NSCLC were obtained from The Cancer Genome Atlas (TCGA) data portal on the UCSC Xena database (https://xenabrowser.net/datapages/) (Goldman et al., 2020). Data of 1,135 tissues (1,027 cancer tissues and 108 para-cancerous tissues) from 1,016 NSCLC patients were obtained.

To obtain the robust and biologically significant DEG list, we extracted DEGs from TCGA-NSCLC–related genes by combining data from lung cancers with different pathological classifications, according to the previous studies (Han et al., 2019; Zhang J. et al., 2020; Zhang et al., 2021). We employed the R package *Deseq2* (Love et al., 2014), set the threshold |log2FoldChange| at >1, and adjusted the p-value at <0.05. NSCLC-related genes were collected by intersecting related genes from public platforms and TCGA data portal. After intersecting the COVID-19–related genes and NSCLC-related genes, we identified genes shared by COVID-19 and NSCLC as interactional genes. These genes might play important roles in pathophysiological processes of COVID-19 and NSCLC and serve as important clues for screening candidate drugs for NSCLC patients with COVID-19.

### Protein–Protein Interaction Network Analysis and Sub-Network Analysis

To interpret associated cellular machinery operations and explore protein mechanisms, PPI network analysis was conducted using STRING (https://string-db.org/) based on proteins derived from COVID-19/NSCLC interactional genes. Active interaction sources of the PPI mainly included text-mining, experiments, databases, co-expression, neighborhood, gene fusion, and co-occurrence. Moreover, to assure the highest confidence of the network, the minimum confidence score was set at 0.90 for network construction. Disconnected nodes were removed from the network. Then Cytoscape 3.9.0 software (Shannon et al., 2003) was applied for network visualization. In the network, the significance of each node was evaluated by betweenness centrality, degree and closeness centrality. In addition, a network analysis module named cytoHubba (Chin et al., 2014) was employed for detecting interactional hub genes. Analytical methods including betweenness, bottleneck, closeness, degree, density of maximum neighborhood component (DMNC), eccentricity, edge-percoalated component (EPC), maximal clique centrality (MCC), maximum neighborhood component (MNC), radiality, and stress were applied to obtain top 10 genes of each method. Based on previous studies, hub genes were defined as genes with a degree value twice the median or more in the whole network (Guo et al., 2015; Yu et al., 2018; Zhuang et al., 2020). Hub genes and genes obtained by the cytoHubba module were intersected, and duplication was removed. By employing these 12 approaches, COVID-19/NSCLC interactional hub genes were identified. Furthermore, sub-network analyses were conducted by the Molecular Complex Detection (MCODE) (Bader and Hogue, 2003) module of Cytoscape 3.9.0 software (Shannon et al., 2003) to identify important gene clusters in the whole PPI network. MCODE is a useful tool for detecting densely connected regions in large protein–protein interaction networks, which helps to identify gene clusters and understand the connectivity and proximity of genes. In the present study, the parameters of MCODE were set as follows: degree cutoff: 2; cluster finding method: haircut; node score cutoff: 0.2; K-Core: 2; and maximal depth: 100.

### Gene Ontology and Kyoto Encyclopedia of Genes and Genomes Pathway Analysis

Based on COVID-19/NSCLC interactional genes, GO analysis and KEGG pathway analyses were carried out by the R package *clusterProfiler* (Wu et al., 2021) to explore potential pathogenic mechanisms of COVID-19 and NSCLC. GO analysis classified associated mechanisms into three categories, including biological processes (BP), cellular components (CC), and molecular functions (MF), and then KEGG pathway analysis further specified related mechanisms. p-value <0.05 was set as a standard metric to quantify the most closely related GO and KEGG terms. Furthermore, to explore the function of interactional genes in the biggest sub-network, GO terms and KEGG pathway terms (p-value < 0.05) relevant to interactional genes were also identified by the R package *clusterProfiler* (Wu et al., 2021)*.*

### Identification of Transcription Factors and MiRNAs Interacting With Interactional Hub Genes

To study underlying regulatory mechanisms at the transcriptional level and identify hub protein’s regulatory molecules, a comprehensive network-based method was employed to decipher regulatory TFs and miRNAs. ChIP-X Enrichment Analysis 3 (ChEA3, https://amp.pharm.mssm.edu/chea3/) (Keenan et al., 2019) is an open-access tool for identifying TFs that are in control of observed alters in gene expression. ChEA3 assembles TFs data from ENCODE (Davis et al., 2018), ReMap (Chèneby et al., 2020), Genotype-Tissue Expression (GTEx) (Carithers and Moore, 2015), ARCHS4 (Lachmann et al., 2018), and Enrichr (Kuleshov et al., 2016). Evidence mainly from ChIP-seq experiments assures the interaction between TFs and customized input genes. The integration method *MeanRank* performed the best in the ChEA3 benchmark and was recommended as the preferable method to present results from different TFs libraries. Results from each library were sorted in ascending order by score using ChEA3. The lower the score, the closer the connection between a gene and a TF. In the study, TFs of COVID-19/NSCLC interactional hub genes were retrieved from ChEA3. Moreover, top 10 TFs with the lowest scores were selected for each gene after removing duplication. Experimentally supported data about the relation of miRNAs to COVID-19/NSCLC interactional hub genes were obtained from Tarbase (Karagkouni et al., 2018) and miRTarbase databases (Chou et al., 2018). To assure the accuracy and robustness of results, we searched the Tarbase database and selected miRNAs that were identified by low-throughput experimental techniques. In the miRTarbase database, the source of miRNAs included a reporter assay, western blot, and qt-PCR. Species was set to *Homo sapiens* in these two databases. Subsequently, regulatory networks of TFs, miRNAs, and COVID-19/NSCLC interactional hub genes were also constructed by Cytoscape 3.9.0 software (Shannon et al., 2003).

### Evaluation of Applicant Drugs for COVID-19 and Non-Small-Cell Lung Cancer

Evaluation of protein–drug interactions is a crucial strategy to detect structural features and respond to receptor sensitivity of proteins, which can pave the way for drug development. To explore promising drugs for COVID-19/NSCLC, drug–proteins interactions were retrieved from the Enrichr database (https://maayanlab.cloud/Enrichr/) (Kuleshov et al., 2016) and the ShinyGO v0.75 web tool (http://bioinformatics.sdstate.edu/go/) (Ge et al., 2020).

Enrichr, containing 192 gene-set libraries and counting, is a comprehensive web portal for gene-set enrichment analysis (Kuleshov et al., 2016). Two series of Enrichr library were downloaded and analyzed, including drug perturbations from GEO (DPFG) and a collection of Drug Signatures Database (DSigDB). DPFG was built based on experimentally supported data from GEO in which gene expression levels were assessed before and after drug administration. Two types of data were retrieved from DPFG for subsequent analyses, including data on the relation of a drug to upregulated genes and data on the relation of a drug to downregulated genes. Species was set to *Homo sapiens* for filtering data. DSigDB is a collection of 22,527 drug-related gene sets with 17,389 compounds covering genes (Yoo et al., 2015). ShinyGO v0.75 is a graphical tool for gene-set enrichment analysis, which is accessible to KEGG and STRING. It applies false discovery rate (FDR) as an adjustment method for p-values of enrichment terms. To obtain robust protein–drug interactions, we merely selected drug molecules which targeted more than half of COVID-19/NSCLC interactional hub genes. Enrichment terms from ShinyGO v0.75 with an adjusted p-value <0.05 were included. Finally, drug molecules obtained from Enrichr libraries and ShinyGO v0.75 were carefully searched on PubChem and PubMed databases. Drug molecules with anticancer and/or antivirus effect were finally selected by browsing relevant articles carefully.

## Result

### Identification of Interactional Genes Between COVID-19 and Non-Small-Cell Lung Cancer

**FIGURE 2:**
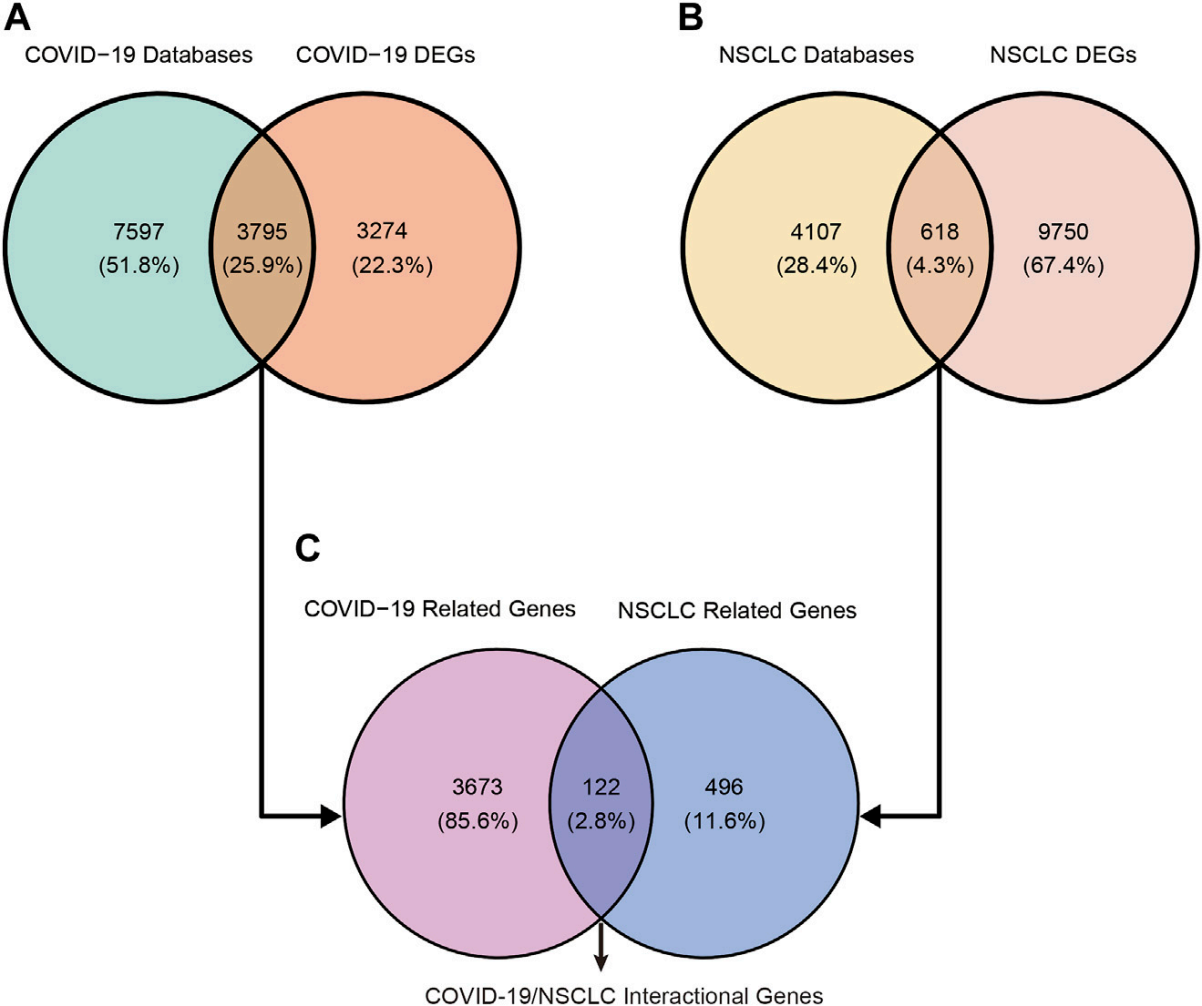
Identification of COVID-19/NSCLC interactional genes by intersecting COVID-19–related genes and NSCLC-related genes from public databases and DEGs from RNA-seq datasets. **(A)** Identification of COVID-19-related genes. **(B)** Identification of NSCLC-related genes. **(C)** Identification of COVID-19/NSCLC interactional genes.

COVID-19–related genes retrieved from OMIM, TTD, PubChem, DisGeNET, GeneCards, and CTD were 2, 78, 628, 1,832, 2,572, and 9,860, respectively (Supplementary File S1). After removing duplication, a total of 11,392 related genes of COVID-19 were obtained from these online databases. COVID-19–related DEGs obtained from the RNA-seq dataset GSE147507, GSE157103, and GSE166190 were 4,825 (3,064 upregulated and 1,761 downregulated), 3,089 (1,422 upregulated and 1,667 downregulated), and 494 (278 upregulated and 216 downregulated), respectively (Table 1; Supplementary File S2). By intersecting COVID-19–related genes from online databases and DEGs from RNA-seq datasets, we obtained 3,795 COVID-19–related genes (Figure 2A).

**TABLE 1:** DEGs in different RNA-seq datasets.

Source	Platform	Group and sample count	DEGs upregulated	DEGs downregulated	DEGs all	Total DEG upregulated	Total DEG downregulated
GSE147507	GPL18573	A549-ACE2 SARS-CoV-2 (6) vs. A549-ACE2 mock (6)	1842	562	2,404	3064	1761
		A549 SARS-CoV-2 (6) vs. A549 mock (13)	225	236	461		
		Calu3 SARS-CoV-2 (3) vs. Calu3 mock (3)	1,293	680	1973		
		COVID-19 lung biopsy (2) vs. Healthy lung biopsy (2)	385	456	841		
		NHBE SARS-CoV-2 (3) vs. NHBE mock (7)	69	49	118		
GSE157103	GPL24676	Female-COVID-ICU (17) vs. female-non COVID-ICU (7)	454	228	682	1,422	1,667
		Female-COVID-non-ICU (21) vs. female-non-COVID-non-ICU (6)	528	539	1,067		
		Male-COVID-ICU (33) vs. male-non-COVID-ICU (8)	587	104	691		
		Male-COVID-non-ICU (29) vs. male-non-COVID-non-ICU (4)	823	1,354	2,177		
GSE166190	GPL20301	Adult-COVID-19-positive vs. Adult-COVID-negative	242	202	444	278	216
		Child-COVID-19-Positive vs.child-COVID-19-negative	40	14	54		
TCGA-NSCLC	Illumina HiSeq	Cancer (1,027) vs. normal (108)	8,229	2,139	10,368	8,229	2,139

NSCLC-related genes collected from OMIM, CTD, TTD, DisGeNET, and GeneCards were 15, 144, 167, 447, and 4,536, respectively (Supplementary File S3). After removing duplication, a total of 4,725 related genes of NSCLC were obtained. A total of 10,368 DEGs (8,229 upregulated and 2,139 downregulated) of NSCLC were identified from the TCGA (Supplementary File S4). By intersecting NSCLC-related genes from online platforms and DEGs from the TCGA, we obtained 618 NSCLC-related target genes (Figure 2B). Finally, a total of 122 COVID-19/NSCLC interactional genes were identified by intersecting COVID-19–related genes and NSCLC-related genes (Figure 2C).

### Systemic Biological Significance of Interactional COVID-19/Non-Small-Cell Lung Cancer Genes

**FIGURE 3:**
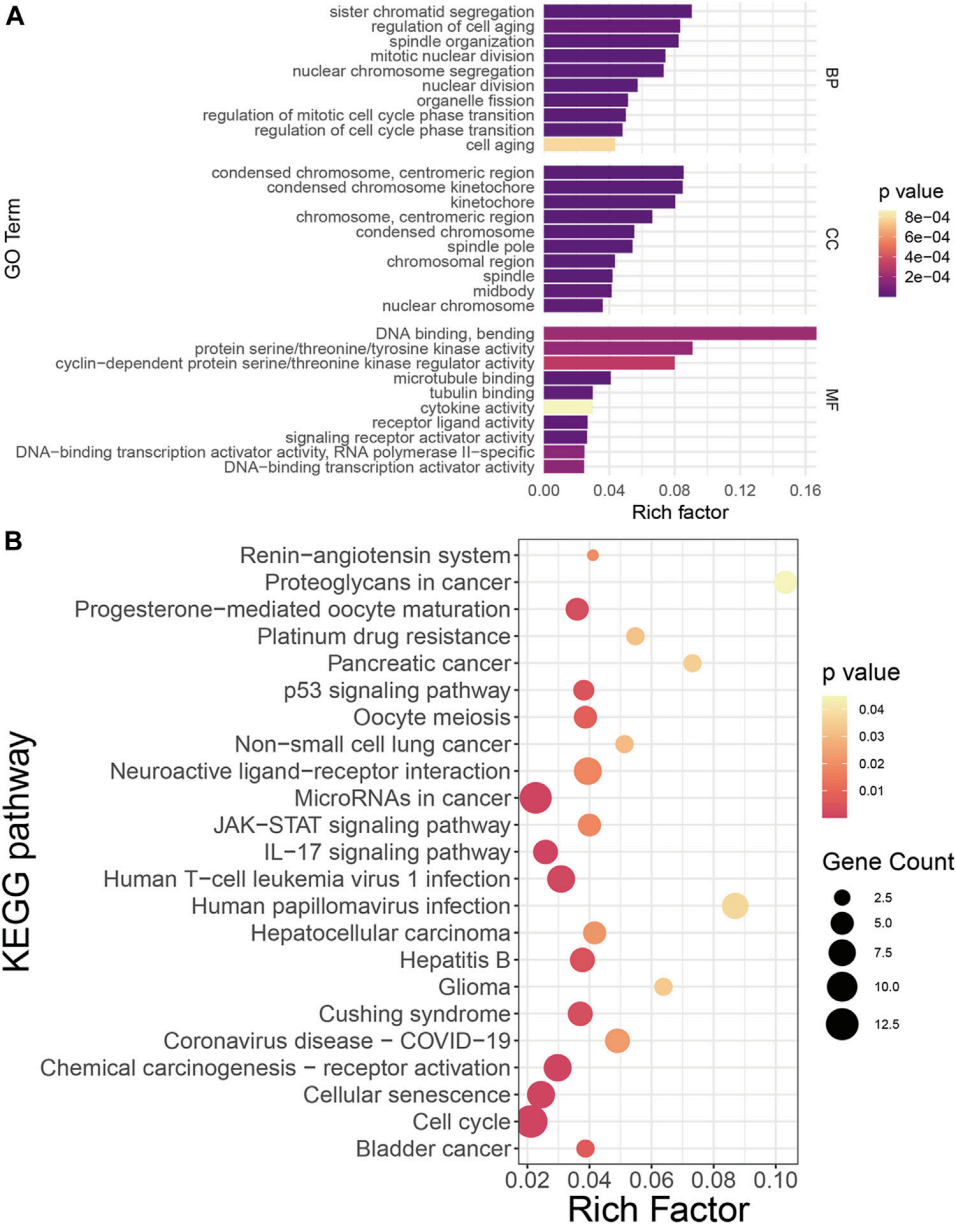
Functional annotation of COVID-19/NSCLC interactional genes. **(A)** GO analysis of COVID-19/NSCLC interactional genes. **(B)** KEGG pathway analysis of COVID-19/NSCLC interactional genes. Note: rich factor is defined as the ratio of input genes that are annotated in a term to all genes that are annotated in this term. The computational formula of rich factor is as follows: Rich factor = number of input genes under this pathway term/number of all annotated genes under this pathway term. The greater the rich factor, the greater the degree of pathway enrichment.

Systemic biology provides multivariate approaches to analyze the larger interactive network of biological pathways holistically and identify important players in disease onset and progression (Starchenko and Lauffenburger, 2018). To reveal characteristics shared by COVID-19 and NSCLC at the genetic level based on systemic biology, GO and KEGG pathway enrichment analyses were performed based on 122 COVID-19/NSCLC interactional genes. As a result, 392 GO terms (BP: 352; CC: 26; and MF: 14) and 23 KEGG pathways were highlighted. The top 10 GO terms of each ontology and 23 KEGG pathways are shown in Figure 3. Representative BP terms included cell aging, regulation of mitotic cell cycle phase transition, and regulation of cell cycle phase transition; representative CC terms included condensed chromosome kinetochore, kinetochore, and condensed chromosome; representative MF terms included cytokine activity, receptor ligand activity, and DNA binding and bending. In addition, representative pathways included the Janus kinase/signal transducers and activators of transcription (JAK/STAT) signaling pathway, interleukin (IL)-17 signaling pathway, chemical carcinogenesis receptor activation, cell cycle, and cellular senescence. These GO terms and KEGG pathways may exert a synergistic effect in the morbidity of COVID-19/NSCLC, which could be clues of therapeutic strategies for these two diseases.

### Identification of Hub Genes and Exploration of Their Interactions

**FIGURE 4:**
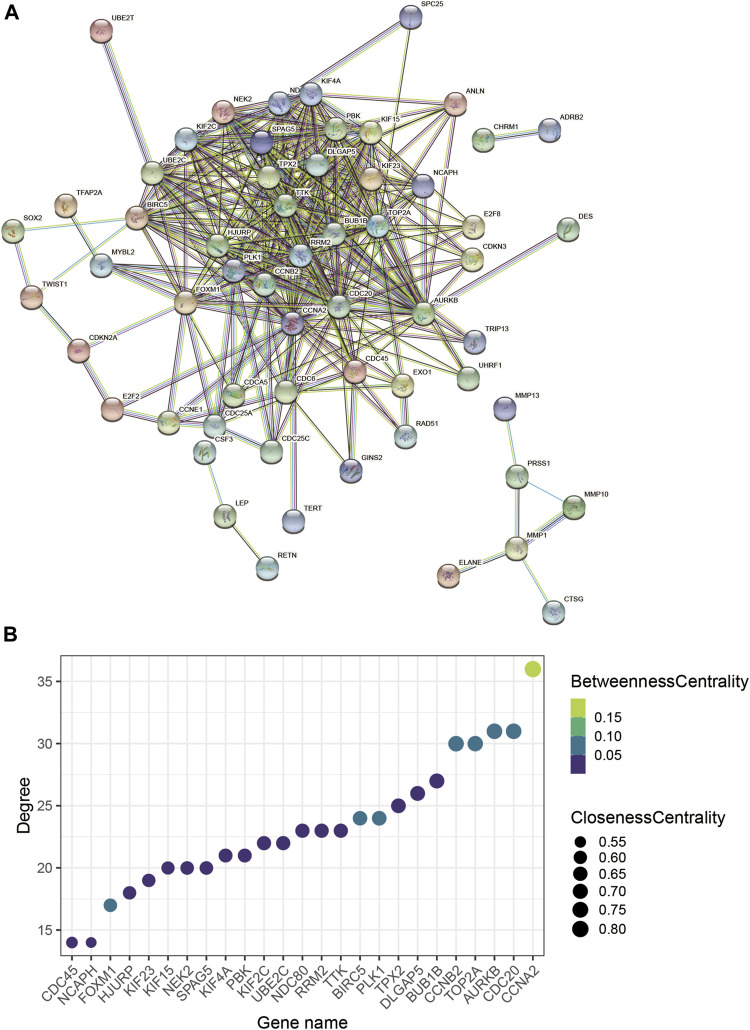
PPI network analysis based on COVID-19/NSCLC interactional genes. **(A)** PPI network containing 59 nodes and 347 edges. **(B)** Bubble chart of the genes with degree value more than the two-fold median degree value in the whole network. Note: nodes represent interactional genes, and edges represent interaction relationships in panel **(A)**.

**FIGURE 5:**
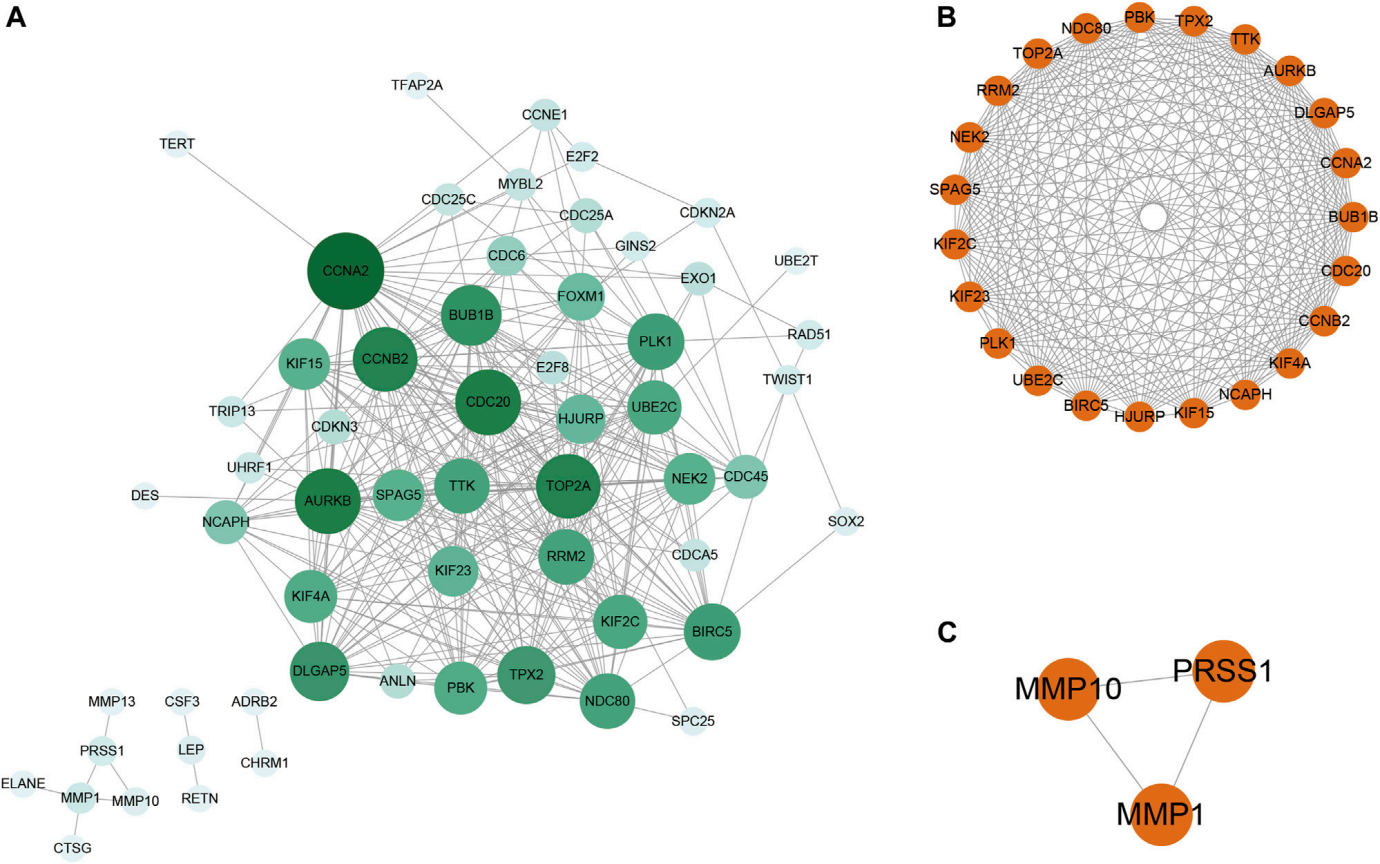
PPI network analysis and sub-network analysis based on identified COVID-19/NSCLC interactional genes. **(A)** PPI network containing 59 nodes and 347 edges. **(B)** Biggest sub-network of the PPI network. **(C)** Small sub-network of the PPI network. Note: nodes represent interactional genes, and edges represent interaction relationships. The depth of the color of the node is positively correlated with the degree value in panel **(A)**.

To explore interrelationships of 122 COVID-19/NSCLC interactional genes, a PPI network containing 59 nodes and 347 edges with the highest confidence scores has been visualized (Figures 4A, 5A). Key parameters of the network were as follows: mean betweenness: 0.05, mean degree: 11.7, and mean closeness: 0.59. A total of 25 DEGs with a degree twice the median or more were identified by the PPI network (Figure 4B). In addition, the other 11 analytical network approaches were employed to identify the top 10 genes for each approach (Table 2). After intersecting genes identified by different approaches, a total of 21 COVID-19/NSCLC interactional hub genes were finally obtained for subsequent analyses. The detail information of these 21 genes is shown in Table 3.

**TABLE 2:** Top 10 important genes identified by different analytical methods.

Method	Gene	Gene count
Betweenness	AURKB, BIRC5, CCNA2, CCNB2, CDC20, FOXM1, MYBL2, PLK1, TOP2A, and UBE2C	10
Bottleneck	BIRC5, CCNA2, CDC20, FOXM1, MYBL2, PLK1, TOP2A, and UBE2C	8
Closeness	AURKB, BIRC5, BUB1B, CCNA2, CCNB2, CDC20, DLGAP5, PLK1, TOP2A, and TPX2	10
Degree	AURKB, BIRC5, BUB1B, CCNA2, CCNB2, CDC20, DLGAP5, PLK1, TOP2A, and TPX2	10
DMNC	BIRC5, HJURP, KIF2C, KIF4A, NCAPH, NDC80, NEK2, SPAG5, TTK, and UBE2C	10
Eccentricity	BIRC5, CCNA2, CCNB2, CDKN3, E2F8, FOXM1, NEK2, PLK1, RAD51, and UHRF1	10
EPC	AURKB, BUB1B, CCNA2, CCNB2, CDC20, DLGAP5, TOP2A, TPX2, TTK, and UBE2C	10
MCC	AURKB, BUB1B, CCNA2, CCNB2, CDC20, DLGAP5, NDC80, TOP2A, TPX2, and TTK	10
MNC	AURKB, BUB1B, CCNA2, CCNB2, CDC20, DLGAP5, RRM2, TOP2A, TPX2, and TTK	10
Radiality	AURKB, BIRC5, BUB1B, CCNA2, CCNB2, CDC20, DLGAP5, PLK1, TOP2A, and TPX2	10
Stress	AURKB, BIRC5, BUB1B, CCNA2, CCNB2, CDC20, FOXM1, PLK1, TOP2A, and UBE2C	10

**TABLE 3:** COVID-19/NSCLC interactional hub genes.

Hub gene	Description	Ensembl gene ID	Entrez	Gene type	Chr	Position (Mbp)
CDC20	Cell division cycle 20	ENSG00000117399	991	Protein coding	1	43.358981
KIF2C	Kinesin family member 2C	ENSG00000142945	11,004	Protein coding	1	44.739818
NEK2	NIMA-related kinase 2	ENSG00000117650	4,751	Protein coding	1	211.658657
RRM2	Ribonucleotide reductase regulatory subunit M2	ENSG00000171848	6,241	Protein coding	2	10.120698
NCAPH	Non-SMC condensin I complex subunit	ENSG00000121152	23,397	Protein coding	2	96.335766
HJURP	Holliday junction recognition protein	ENSG00000123485	55,355	Protein coding	2	233.833416
CCNA2	Cyclin A2	ENSG00000145386	890	Protein coding	4	121.816444
TTK	TTK protein kinase	ENSG00000112742	7,272	Protein coding	6	80.003887
FOXM1	Forkhead box M1	ENSG00000111206	2,305	Protein coding	12	2.85768
DLGAP5	DLG-associated protein 5	ENSG00000126787	9,787	Protein coding	14	55.148112
BUB1B	BUB1 mitotic checkpoint serine/threonine kinase B	ENSG00000156970	701	Protein coding	15	40.161023
CCNB2	Cyclin B2	ENSG00000157456	9,133	Protein coding	15	59.105126
PLK1	Polo-like kinase 1	ENSG00000166851	5,347	Protein coding	16	23.677656
AURKB	Aurora kinase B	ENSG00000178999	9,212	Protein coding	17	8.204733
SPAG5	Sperm-associated antigen 5	ENSG00000076382	10,615	Protein coding	17	28.577565
TOP2A	DNA topoisomerase II alpha	ENSG00000131747	7,153	Protein coding	17	40.388525
BIRC5	Baculoviral IAP repeat-containing 5	ENSG00000089685	332	Protein coding	17	78.214186
NDC80	NDC80 kinetochore complex component	ENSG00000080986	10,403	Protein coding	18	2.571557
TPX2	TPX2 microtubule nucleation factor	ENSG00000088325	22,974	Protein coding	20	31.739271
UBE2C	Ubiquitin-conjugating enzyme E2 C	ENSG00000175063	11,065	Protein coding	20	45.812576
KIF4A	Kinesin family member 4A	ENSG00000090889	24,137	Protein coding	X	70.290104

**FIGURE 6:**
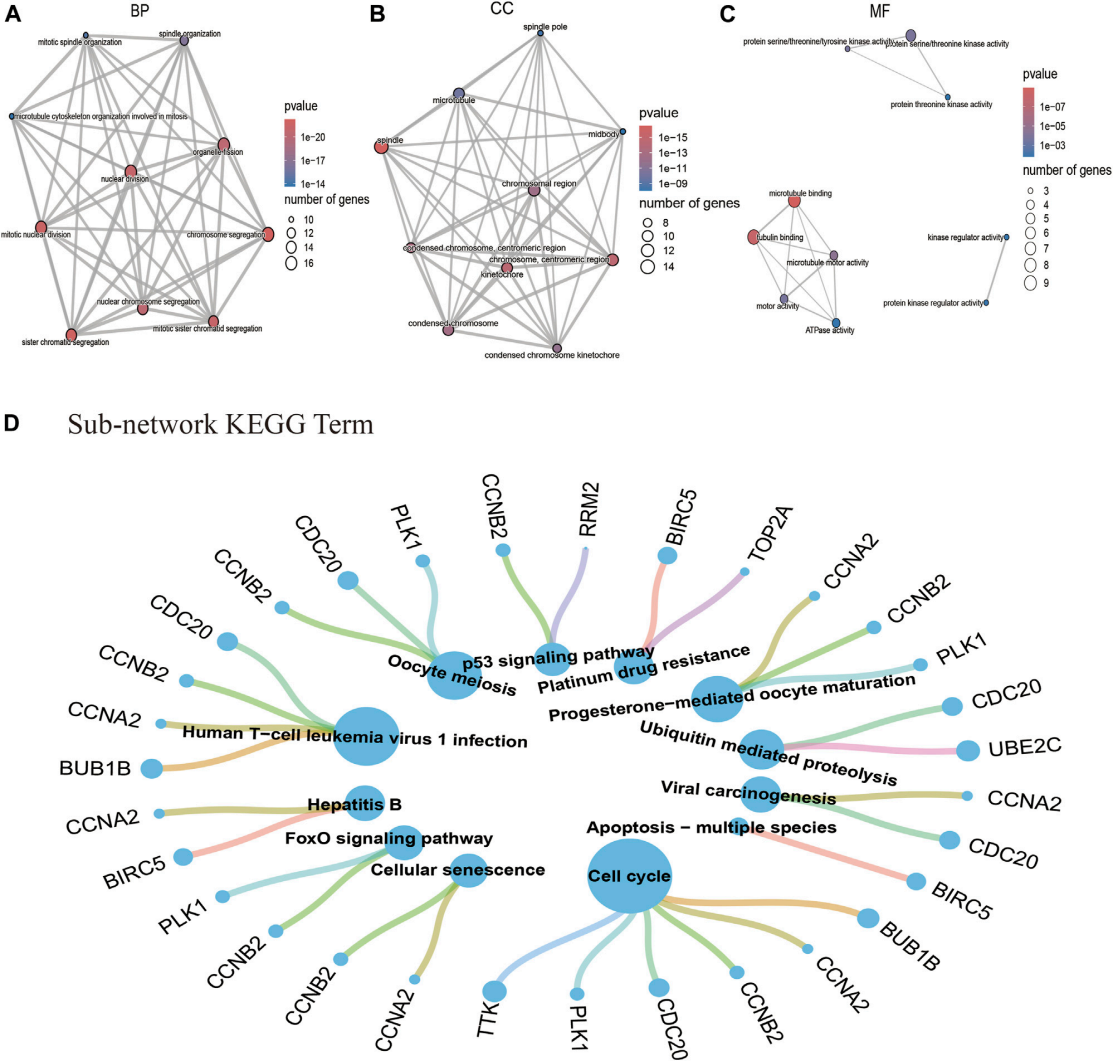
GO and KEGG pathway analysis based on the interactional genes in the biggest sub-network. **(A)** Top 10 BP terms of GO analysis. **(B)** Top 10 CC terms of GO analysis. **(C)**Top 10 MF terms of GO analysis. **(D)** Twelve pathway terms of KEGG analysis. Note: nodes represent genes or pathways, and edges represent enrichment relationships. The size of term nodes is positively correlated with the number of enriched genes. The size of gene nodes is positively correlated with the number of enriched terms.

To further understand the main systemic biological significance of the network, a sub-network analysis based on nodes of the whole network was conducted. With the recommended parameters in the molecular complex detection (MCODE) module (degree cutoff: 2, node score cutoff: 0.2, K-Core: 2, and maximum depth = 100), two sub-networks were extracted from the primary one (Figures 5B,C). Based on the biggest sub-network containing 23 nodes and 232 edges (Figure 5B), 283 GO terms (BP: 235; CC: 35; and MF: 13) and 12 KEGG pathways were highlighted (Supplementary File S5). The top 10 terms of each GO and all the KEGG pathways are shown in Figures 6A–D. Typical BP terms mainly involved mitotic spindle organization, organelle fission, and chromosome segregation; typical CC terms mainly involved chromosomal region, midbody, and spindle; typical MF terms mainly involved protein threonine kinase activity, microtubule binding, and kinase regulator activity. In addition, the main KEGG pathways involved cell cycle, viral carcinogenesis, p53 signaling pathway, and cellular senescence. By comparing the KEGG pathways from the whole PPI network and the main sub-network, functions of COVID-19/NSCLC interactional genes in cell cycle, cellular senescence, and p53 signaling pathway were underlined.

### Determination of Regulatory Signatures

**FIGURE 7:**
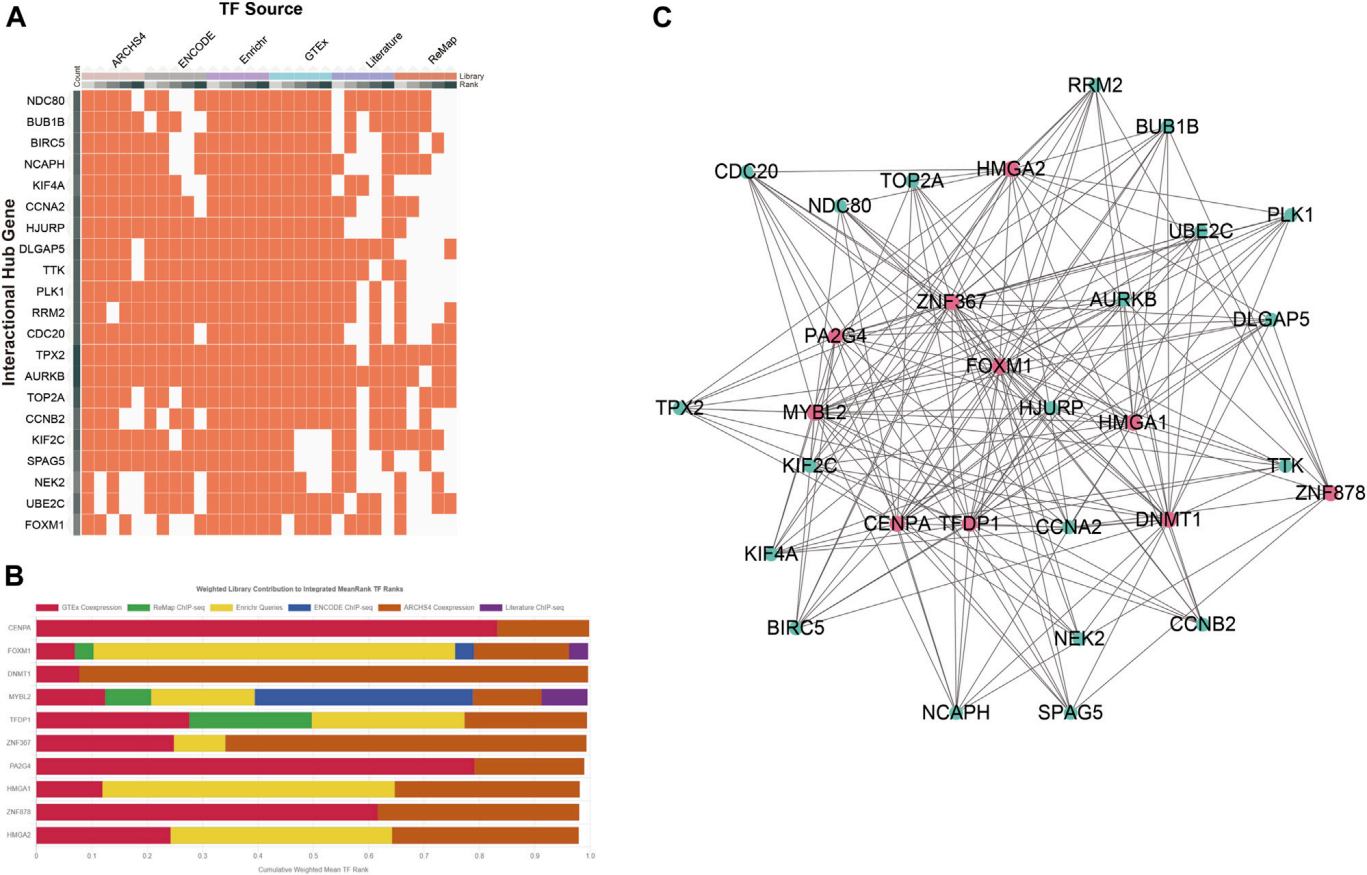
Determination of regulatory signatures (TFs). **(A)** Data source of TFs of the COVID-19/NSCLC interactional hub gene. **(B)** Top 10 TFs identified by the ChEA3 database. **(C)** TFs–interactional hub genes interaction network. Note: red nodes represent TFs, green nodes represent genes, and edges represent interaction relationships in panel **(C)**.

A network-based approach was employed to identify regulatory TFs and miRNAs and to have a glimpse of changes happening at the transcriptional level. A total of 1,632 TFs were filtered from the ChEA3 database and sorted by the *MeanRank* method for the most robust result (Supplementary File S6). The data source of TFs of each COVID-19/NSCLC interactional hub gene is shown in Figure 7A. The top 10 TFs of the interactional hub gene recommended by the ChEA3 database were retrieved for subsequent analyses (Figure 7B). Next, a TF–gene interaction network containing 30 nodes and 186 edges was constructed (Figure 7C). Centromere protein A (CENPA), DNA methyltransferase 1 (DNMT1), MYB proto-oncogene-like 2 (MYBL2), transcription factor Dp-1 (TFDP1), and zinc finger protein 367 (ZNF367) were identified as the most influential regulatory factors since they targeted all interactional hub genes.

**FIGURE 8:**
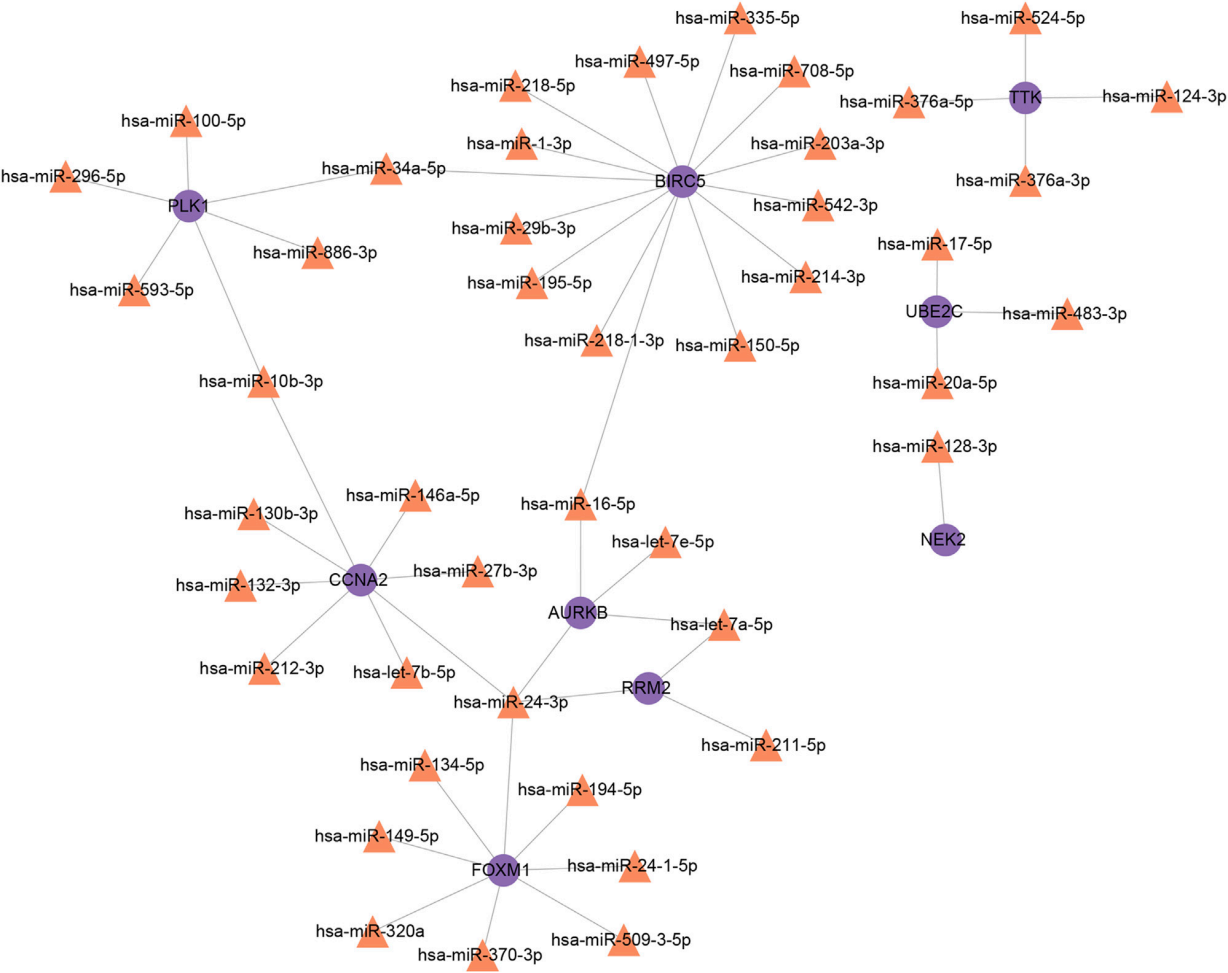
MiRNAs–interactional hub genes interaction network. Note: purple nodes represent genes, orange nodes represent MiRNAs, and edges represent the interactions between nodes.

Furthermore, we obtained 17 and 40 miRNAs from Tarbase and mirTarbase, respectively (Supplementary File S7). After removing duplicated outcomes, a total of 44 miRNAs engaged with interactional hub genes of COVID-19 and NSCLC were identified. A miRNA–gene interaction network comprising 53 nodes and 51 edges was built (Figure 8). Representative miRNAs included hsa-miR-24-3p (targeting at four interactional hub genes), hsa-miR-16-5p (targeting at two interactional hub genes), hsa-let-7a-5p (targeting at two interactional hub genes), hsa-miR-34a-5p (targeting at two interactional hub genes), and hsa-miR-10b-3p (targeting at two interactional hub genes). Taken together, these two networks indicated that 10 important TFs and 44 post-transcriptional regulatory signatures (miRNAs) provided evidence for exploring regulatory mechanisms of COVID-19 and NSCLC by participating in the regulation of interactional hub genes.

### Identification of Candidate Drugs for COVID-19 and Non-Small-Cell Lung Cancer

To identify prospective agents for COVID-19 and NSCLC, we selected potential candidates from the Enrichr database, including DSigDB, DPFG, and ShinyGO v0.75. Studies of drug molecules from these three sources were retrieved from the PubMed database for screening drugs with anticancer and/or antivirus effect (Supplementary Table S1). According to the result, the number of drug molecules obtained from DSigDB, DPFG, and ShinyGO v0.75 was 15, 10, and 3, respectively. A total of 23 candidates were identified after removing duplication. Among them, nine drugs have already been used in NSCLC, while six drugs have been registered to clinical trials for COVID-19. Resveratrol was identified both in DSigDB and DPFG, lucanthone was identified both in DSigDB and ShinyGO v0.75, and vemurafenib was identified both in DPFG and ShinyGO v0.75. Notably, natural small molecules included resveratrol and quercetin worth focus because of their binding affinity with molecular ACE-2.

## Discussion

The present outbreak of SARS-CoV-2 has spread to 224 countries, with more than 250 million people infected (Worldometer, 2021). The elderly and those with pre-existing complications or smoking-related lung damage are susceptible to COVID-19, while they are precisely the profile of lung cancer patients (Berlin et al., 2020; Liao et al., 2020). The emergence of COVID-19 has resulted in detrimental alterations including (epi-)genetic alterations and changes at the transcriptional level and related signaling pathways, which have changed lung cancer treatment provision (Alnajeebi et al., 2021). Various candidate drugs and vaccines are being studied for the treatment of COVID-19; nevertheless, no therapy has shown a specific effect (Majumder and Minko, 2021). Therefore, in this study, we conducted a comprehensive systemic biological and bioinformatic analysis to identify potential interactional biomarkers that might help understand co-pathogenic factors of these two diseases and provide clues for the treatment of COVID-19 and NSCLC.

### Identified Related Genes Indicated Genetic Interrelationships of COVID-19 and Non-Small-Cell Lung Cancer

Bioinformatic and systemic biological analysis can integrate data of gene expressions and protein interactions, analyze gene-regulatory pathways, and construct protein–protein networks, which is an effective tool for molecular mechanisms (Durmuş et al., 2015; Ahmed, 2020) and drug development (van Driel and Brunner, 2006), especially in the field of cancer treatment (Lee et al., 2017; Wooller et al., 2017) and infectious diseases (Josset et al., 2010; Li J. et al., 2016). NSCLC is a genomic disease which involves a loss of control over vital cellular functions and could be deteriorated by COVID-19 infection due to differential expressions of multiple host genes (Ahmed, 2020). Biomarker identification is helpful in managing NSCLC or COVID-19 (Samprathi and Jayashree, 2020; Wang et al., 2021). Inherent cancer-related changes in NSCLC genome and the aberrant expression of host factors caused by COVID-19 infection could interact with each other and impact the prognosis of patients (Passaro et al., 2021). In this study, we identified 122 COVID-19/NSCLC interactional genes through bioinformatic and systemic biological approach, which would pave the way to reveal common pathophysiological mechanisms shared by COVID-19 and NSCLC (Ahmed, 2020; Samprathi and Jayashree, 2020; Wang et al., 2021).

### Gene Ontology and Kyoto Encyclopedia of Genes and Genomes Analyses Highlighted Systemic Biological Significance

To elucidate the roles of the identified 122 COVID-19/NSCLC interactional genes, GO and KEGG pathway analyses were conducted. In GO analysis, biological processes were mostly enriched in sister chromatid segregation, regulation of cell aging, spindle organization, and mitotic nuclear division. Dysregulated cell–cycle machinery, uncontrolled proliferation, and resistance to programmed cell death feature cancers, including NSCLC (Sherr, 1996; Hanahan and Weinberg, 2000; Smolle et al., 2019). Due to the paucity of well-proven therapies or vaccines, inhibiting cell division may be a good way to control COVID-19, which shares a similar goal with NSCLC treatment (Borcherding et al., 2020). Some of interactional genes participated in the BP have been found to be closely associated with the development and prognosis of NSCLC and COVID-19. For example, aurora kinase B (*AURKB*) has been found among the DEGs of SARS-CoV-2 in Caco-2 cells (Bock and Ortea, 2020). In addition, an *in vitro* experiment suggested that *AURKB* inhibition caused cell cycle arrest and polyploidy, followed by extensive cell death in the NSCLC model (Bertran-Alamillo et al., 2019). During SARS-CoV-2 infection, the *PLK1* gene and its translational products performed significant tasks in the normal cell division cycle, promoting the spread of virus (Chen et al., 2021). The inhibition of *PLK1* could alter the immune microenvironment of NSCLC by increasing dendritic cell maturation and enriching T-cell infiltration (Zhou et al., 2021).

KEGG links a group of genes in the genome with the higher-order functional information *via* computerizing current knowledge about cellular processes and standardizing gene annotations (Kanehisa and Goto, 2000). Based on KEGG pathway analysis, we found pathways shared by COVID-19 and NSCLC, including cell cycle, celluar senescence, IL-17 signaling pathway, chemical carcinogenesis-receptor activation, p53 signaling pathway, and Janus kinase/signal transducer and activator of the transcription (JAK-STAT) signaling pathway. Previous studies suggested that JAK-STAT, IL-17, and p53 signaling pathways were not only closely associated with the tumorigenesis of NSCLC *via* regulating cell proliferation, differentiation, and apoptosis but also involved in the promotion of cytokine storm which deteriorated conditions in COVID-19 patients (Shibabaw, 2020; Hu H. et al., 2021; Satarker et al., 2021). Regulated by interferon (IFN) signaling, the endocytosis of SARS-CoV-2 in lung epithelial cells is initiated by the ACE2 receptor. A cross-sectional study showed that in severe COVID-19 patients, dysregulated type I/III IFNs and JAK/STAT signaling caused impaired antiviral responses (Hadjadj et al., 2020). During the development of COVID-19, SARS-CoV-2 infection induced an excessive immune response and released a variety of pro-inflammatory cytokines through the JAK/STAT pathway, such as IL-2, IL-6, and granulocyte colony-stimulating factor signaling (Solimani et al., 2021). In addition, high levels of pro-inflammatory cytokines and chemokines accelerated disease progression, thus becoming the main risk factor of cellular senescence and age-related diseases (Bruunsgaard et al., 2003; Hu X. et al., 2021). Previous studies showed that the severity of COVID-19 and NSCLC was closely associated with hyperinflammation that could drive lung and multiorgan injury and increase mortality *via* cytokine storm and sepsis (Gomes et al., 2014; Tay et al., 2020). Thus, targeting inflammation-related signaling pathways may be a good option for the treatment of COVID-19 and NSCLC.

Among the interactional genes involved in the aforementioned pathways, cyclin A2 (*CCNA2*) and cyclin B2 (*CCNB2*) are members of the cyclin protein family, which play critical roles in controlling cell cycle, cell senescence, and viral infection (Nam and van Deursen, 2014). Overexpressed *CCNA2* and *CCNB2* are correlated with a poor prognosis in NSCLC (Cooper et al., 2009; Takashima et al., 2014; Qian et al., 2015). Meanwhile, during SARS-CoV-2 infection, the increasing level of *CCNA2* not only provides favorable conditions for virus transmission but also hints at chromosomal abnormalities and other genetic material damage in the host cells (Chen et al., 2021). Cellular senescence, an irreversible state of cell cycle arrest in response to damaging stimuli, secretes factors known as senescence-associated secretory phenotype or SASP (González-Gualda et al., 2021; Kumari and Jat, 2021). In this case, cells maintain active metabolism without responses of mitogenic and apoptotic signals (González-Gualda et al., 2021). In the elderly or patients upon consistent and chronic damages, cellular senescence may impair regenerative ability and accelerate the progression of inflammation and lung cancer (Hernandez-Segura et al., 2018; Kuźnar-Kamińska et al., 2018). In lung cancer tumorigenesis, senescent cells produced soluble signaling factors [including interleukins (Rabinovich et al., 2007), chemokines (Coppé et al., 2010), and growth factors (Yang et al., 2006)], proteases (Finkel et al., 2007), insoluble proteins, and extracellular matrix components (Acosta et al., 2008a; Acosta et al., 2008b), which mediated cell proliferation, invasion, and migration (Wald et al., 2011; Han et al., 2015; Kuźnar-Kamińska et al., 2016). Furthermore, a strong connection between cellular senescence and SARS-CoV-2 has been found, as cellular senescence increased the risk of developing severe COVID-19 (Nehme et al., 2020). SARS-CoV-2 infection could induce paracrine senescence by increasing IFN secretion in infected cells, and danger-associated molecular patterns released in cells undergoing pyroptosis or necroptosis further accelerated senescence in the environment *via* SASP factors (Kim et al., 2009; Acosta et al., 2013; Kandhaya-Pillai et al., 2017; Nehme et al., 2020). Cellular senescence, in turn, 1) led to a weak adaptive immunity by increasing senescent-like T cells and B lymphocytes (Frasca et al., 2017; De Biasi et al., 2020); 2) enhanced aberrant healing response and tissue fibrosis in the respiratory system (Mason, 2020); 3) resulted in vascular dysfunction *via* decreasing angiogenesis and increasing thrombosis and inflammatory responses in COVID-19 patients (Ungvari et al., 2018; Escher et al., 2020). Dysregulation of the cell cycle and cellular senescence has been observed in both COVID-19 and lung cancer. Thus, the importance of understanding the homeostasis maintenance and pathological alteration of these biological processes should be emphasized.

In summary, the aforementioned genes and pathways are commonly and significantly altered in both COVID-19 and NSCLC, preparing the ground for pathophysiological studies and drug development.

### Protein–Protein Interaction and Sub-Network Analysis Explored In-Depth Interrelationships of Interactional Hub Genes

On the basis of interactional genes, we built a PPI network presenting in-depth biological characteristics. In the biggest sub-network, *CCNA2*, *CCNB2*, *AURKB*, DNA topoisomerase II alpha (*TOP2A*), and baculoviral IAP repeat containing 5 (*BIRC5*) were closely connected with other genes. *TOP2A*, a target for cytotoxic drugs (etoposide, anthracyclines) and a key regulator of chromosome condensation and chromatid separation, prevents DNA replication and transcription (Nicoś et al., 2021). It is generally believed that *TOP2A* is a prognostic indicator of NSCLC, and its level is negatively correlated with the prognosis (Hou et al., 2017; Ma et al., 2019). In consistence with the result of our study, *TOP2A* has been identified as a hub gene that could govern many cellular processes by protein–protein interactions in COVID-19 patients (Hasan et al., 2022).

Comparing to the result of KEGG analysis based on 122 interactional genes, the biggest sub-network identified additional pathways, such as forkhead box, sub-group O (FoxO) signaling pathway, ubiquitin-mediated proteolysis, viral carcinogenesis, and apoptosis. As tumor suppressors, FoxO proteins increased the expression level of death receptor ligands such as Fas ligand and tumor necrosis factor (TNF) apoptosis ligand, engaged with pro-apoptotic pathways, and blocked cell cycle progression (Farhan et al., 2017). According to the result, viral carcinogenesis was one of the most important KEGG pathways, indicating that co-pathogenic factors shared by COVID-19 and NSCLC could be the basis of finding potential drugs for synergistic treatment.

### Interaction of Transcription Factors and MicroRNAs With Interactional Hub Genes

Genetic alterations have long been blamed for malignancy but cannot fully explain tumor development. Epigenetic dysregulations give new insights into how heritable changes in gene expression happen without involving changes in nucleotide sequence, and finally promote carcinogenesis (Yuan et al., 2016). MiRNAs and TFs are the largest families of trans-acting gene regulatory species and pivotal players in a complex regulatory network (Sharma et al., 2020). Herein, we investigated TF–gene and miRNA–genes interaction that might help in learning more about disease development.

Transcription factors are key cellular components that regulate multiple genes over a long distance by maintaining proliferation status, driving cellular differentiation, and determining cell fate (Essebier et al., 2017). The identified top 10 TFs included CENPA, DNMT1, MYBL2, TFDP1, ZNF367, high mobility group AT-hook 1 (HMGA1), proliferation-associated 2G4 (PA2G4), high mobility group AT-hook 2 (HMGA2), zinc finger protein 878 (ZNF878), and FOXM1. Among them, CENPA (Mullen et al., 2020), DNMT1 (Wu et al., 2020), MYBL2 (Mullen et al., 2020), TFDP1 (Zhan et al., 2017), ZNF367 (Liu Z. et al., 2018), HMGA1 (Zhang Z. et al., 2015), HMGA2 (Gao et al., 2018), and FOXM1 (Mullen et al., 2020) were previously shown to be highly associated with NSCLC progression. It has been demonstrated that CENPA, MYBL2, and FOXM1 were linked to numerous cancer-specific enhancers, and their elevated expression levels were associated with a poor survival rate of NSCLC patients (Mullen et al., 2020). Meanwhile, the level of DNMT1 was found significantly downregulated in SARS-COV-2–infected epithelial cells (Muhammad et al., 2021). SARS-COV-2 infection mainly affects the molecular mechanisms of aging centered on HMGA1 and HMGA2 proteins, and their interactions may impair or trigger inflammatory pathways, leading to various responses in different age groups (Mercatelli et al., 2021). To conclude, DNMT1, HMGA1, and HMGA2 might be central TFs in the TF–gene regulatory network in COVID-19/NSCLC.

MiRNAs are endogenous small non-coding RNA molecules that can regulate the expression of non-coding sequences and genes involved in oncogenesis (Liu et al., 2014). Various studies have identified miRNAs as key players in the pathogenesis and therapeutics of viral diseases. Moreover, as part of host–pathogen interactions, miRNA can scan target SARS-CoV-2 genes as well as host inflammatory machinery to counter-act the impairing effects of infection (Ghosh et al., 2009; Khokhar et al., 2022). The top 10 most significant miRNAs mainly involved in respiratory diseases (Table 4), including NSCLC [hsa-miR-24-3p (Wei et al., 2021), hsa-miR-34a-5p (Zhou X. et al., 2020), hsa-miR-10b-3p (Liu et al., 2017), hsa-miR-20a-5p, and hsa-miR-17-5p (Leidinger et al., 2016)], idiopathic pulmonary fibrosis [hsa-miR-524-5p (Li Q. et al., 2020), COVID-19 (hsa-miR-20a-5p, hsa-miR-17-5p (Li C. et al., 2020)), and hsa-miR-16-5p (Kim et al., 2020)], pneumoconiosis [hsa-let-7a-5p (Zhang et al., 2018)], and asthma [(hsa-let-7a-5p (Huang et al., 2019)]. Interestingly, compared with the healthy controls, the levels of hsa-miR-20a-5p and hsa-miR-17-5p were significantly downregulated in patients with COVID-19; therefore, they were considered as essential modulators of viral replication (Li C. et al., 2020). In addition, according to a study based on high-throughput qRT-PCR validation, hsa-miR-20a-5p and hsa-miR-17-5p were the top markers that could distinguish NSCLC patients from unaffected controls with 94.5% accuracy (Leidinger et al., 2016).

**TABLE 4:** Top 10 miRNAs involved in various respiratory diseases.

MiRNA name	Respiratory disease type	References
hsa-miR-24-3p	NSCLC	Wei et al. (2021)
hsa-miR-16-5p	COVID-19	Kim et al. (2020)
hsa-let-7a-5p	Pneumoconiosis and asthma	Zhang et al. (2018), Huang et al. (2019)
hsa-miR-34a-5p	NSCLC	Xingni Zhou et al. (2020)
hsa-miR-10b-3p	Pneumoconiosis and asthma	Liu et al. (2017)
hsa-miR-20a-5p	NSCLC and COVID-19	Leidinger et al. (2016), Caixia Li et al. (2020)
hsa-miR-17-5p	NSCLC and COVID-19	Leidinger et al. (2016), Caixia Li et al. (2020)
hsa-miR-524-5p	Idiopathic pulmonary fibrosis	Qilong Li et al. (2020)
hsa-miR-376a-5p	Has not been reported	Not available
hsa-miR-483-3p	Has not been reported	Not available

Notably, hsa-miR-24-3p, hsa-miR-16-5p, and hsa-let-7a-5p targeted *AURKB*, central regulator of cell division (Bertran-Alamillo et al., 2019), indicating that these miRNAs may be potential targets to control cell cycle and cellular senescence. In addition, miR-34a-5p regulated the G1/S checkpoint in NSCLC cells (Gupta et al., 2020). Forced expression of miR-34a-5p enhanced p21 expression and promoted cellular senescence, whereas downregulated miR-34a-5p decreased senescence and increased apoptosis by targeting B-cell lymphoma-2 (BCL2), myelocytomatosis oncogene (MYC), mesenchymal-epithelial transition (MET), and p53 (Gallardo et al., 2009; Kasinski and Slack, 2012). According to the result, *BIRC5*, *FOXM1*, *CCNA2*, and *PLK1* showed a higher degree of interaction with miRNAs, which may play crucial roles in the interaction networks.

To sum up, TF–gene intereactions are reactors that regulate gene expression by binding with target genes and miRNAs (Zhang HM. et al., 2015). The constructed TF–gene and miRNA–gene interaction networks help further understand the direct regulatory relationship of miRNA and TFs in NSCLC and COVID-19, while interactional hub genes may be crucial biomarkers and therapeutic targets.

### Potential Drugs Provided Possible Treatments for COVID-19 and Non-Small-Cell Lung Cancer

In this study, a total of 23 candidates containing 18 drugs and 5 natural compounds were identified. Among them, nine drugs have already been administered to NSCLC patients, including irinotecan (Murakami et al., 2018), dasatinib (Xu et al., 2015), 5-fluorouracil (Nokihara et al., 2017), etoposide (Liang et al., 2017), carboplatin (Hasegawa et al., 2020), ascorbic acid (Pathak et al., 2005), azacitidine (Cheng et al., 2021), decitabine (Chu et al., 2013), and palbociclib (Nie et al., 2019). According to the ClinicalTrials database (https://clinicaltrials.gov/), cyclosporin A (NCT04540926), dasatinib (NCT04830735), quercetin (NCT04851821), etoposide (NCT04356690), ascorbic acid (NCT04401150), and decitabine (NCT04482621) have been registered to clinical trials for evaluating their efficacy in COVID-19 patients. Therefore, it can be inferred that dasatinib, etoposide, ascorbic acid, and decitabine are likely to be the promising agents for NSCLC patients with COVID-19, which provides support for our predictions.

Quercetin and resveratrol are two natural compounds which have both antiviral and anti-NSCLC effects (Zhao et al., 2010; Wu et al., 2015; Lin et al., 2017; Chai et al., 2021). Quercetin, a well-known natural polyphenol with anti-inflammatory, antioxidant, and immunomodulatory properties, is involved in a variety of diseases, such as viral infections, respiratory diseases, allergies, asthma, and cancer (Li Y. et al., 2016; Xu et al., 2019). Previous network pharmacology and molecular docking studies anticipated that quercetin could interfere with SARS-CoV-2 replication by interacting with 3-chymotrypsin-like protease (3CLpro), papain-like protease (PLpro), and S proteins (Derosa et al., 2021; DI Pierro et al., 2021b). An open-label, randomized controlled trial included 152 outpatients with confirmed SARS-CoV-2 infection but without severe COVID-19 symptoms found that formulated quercetin treatments reduced the frequency and length of hospitalization, the need of non-invasive oxygen therapy, and the number of deaths (DI Pierro et al., 2021a). In addition, available experimental studies suggested that quercetin could directly modulate multiple lung cancer-relevant miRNAs and DNA methylation (Kim DH. et al., 2019; Kedhari Sundaram et al., 2019). Another ingredient resveratrol is a potent antioxidant that could inhibit platelet aggregation and vasodilation, reduce blood viscosity, and maintain blood flow (Olas and Wachowicz, 2005; de la Lastra and Villegas, 2007). *In vitro* and *in vivo* studies indicated that resveratrol induced cell apoptosis and inhibited proliferation, growth, and metastasis in NSCLC (Yousef et al., 2017; Tang et al., 2020). Given the thrombin-inhibitory and anti-inflammatory effects, resveratrol deserves further study for the treatment of COVID-19 and NSCLC.

It is noteworthy that a computational structure-based study identified trichostatin A as a potential SARS-CoV-2 Mpro inhibitor. The result has been further validated by an essay which has suggested that trichostatin A could reduce the viral RNA load, viral antigen expression, and infectious virus particle formation (Wen et al., 2021). Other identified ingredients also had an anti-respiratory virus effect. For instance, cyclosporine at non-cytotoxic concentrations could induce a strong inhibition of the replication of specific coronaviruses *in vitro*, including SARS-CoV, MERS-CoV, and human coronavirus 229E (HCoV-229E) (Poulsen et al., 2020). A retrospective observational study recently found that in COVID-19 hospitalized patients, cyclosporine was significantly associated with a decrease in mortality, probably due to its combined activity of immunosuppression and antiviral activity (Guisado-Vasco et al., 2020). Dyall et al. (2014) found that dasatinib was active against both MERS-CoV and SARS-CoV *in vitro*, and it might minimize immunotoxicity as it blocked viral replication. Vemurafenib interfered the cellular Raf/MEK/ERK signaling cascade by binding to the ATP-binding site of BRAF (V600E) kinase and inhibiting its function (Pleschka et al., 2001). Interestingly, since Raf/MEK/ERK signaling pathways mediated the increasing SARS-CoV-1 replication (Dyall et al., 2014), it may be a therapeutic target for host-directed SARS-CoV-2 antivirals. Thus, vemurafenib may also be a potential anti-COVID-19 drug. To conclude, according to available literature and the result of our study, identified molecules may be prospective agents in the treatment of COVID-19 and NSCLC.

Based on the comprehensive bioinformatic and systemic biological analysis, the present study carried out a study framework to reveal interaction networks and therapeutic implications for NSCLC patients with COVID-19, the findings of which would provide evidence and shed light for the further research on COVID-19/NSCLC. However, there were still some limitations in the present study. First, the sample size of certain disease studies might be insufficient to capture all of the critical disease-related genes for identifying the common DEGs. Second, incompleteness of available interactome data and limitation of computational methods might make the conclusions less dependable and accurate. Finally, the results of the present study were derived from multiple computational approaches, and future *in vivo* and *in vitro* experiments are required to fully assess the biological relevance of candidates.

## Conclusion

In summary, based on bioinformatic analyses, we predicted promising therapeutic ingredients and drugs for NSCLC patients with COVID-19. We detected interactional hub genes enriched in regulating biological processes and signaling pathways, which were mainly relevant to cell division, cell aging, cell cycle, and cellular senescence. This study has identified 1) potential therapeutic targets, including *CCNA2*, *CCNB2*, *AURKB*, *TOP2A*, and *BIRC5*; 2) signaling pathways primarily related to cell cycle, cell aging, viral carcinogenesis, and p53 signaling pathway; 3) potential agents for the treatment of COVID-19 and NSCLC, including quercetin, resveratrol, cyclosporine, dasatinib, etoposide, ascorbic acid, and decitabine. Future experimental and clinical studies should be carried out with predicted agents to explore pharmacological mechanisms and to inform possible interventions for COVID-19 and NSCLC.

## Data Availability

The datasets presented in this study can be found in online repositories. The names of the repository/repositories and accession number(s) can be found in the article/Supplementary Material.

## Supplementary Material

Supplementary File S1 COVID-19–related genes obtained from public databases.

Supplementary File S2 COVID-19–related DEGs obtained from the RNA-seq dataset.

Supplementary File S3 NSCLC-related genes obtained from public databases.

Supplementary File S4 DEGs of NSCLC obtained from TCGA database.

Supplementary File S5 GO and KEGG terms based on the biggest sub-network with the associated statistics.

Supplementary File S6 TFs filtered from the ChEA3 database.

Supplementary File S7 MiRNAs obtained from Tarbase and mirTarbase.

Supplementary Table S1 Antiviral and/or anticancer activities of candidate drugs for COVID-19/NSCLC from Enrichr database and ShinyGO v0.75 database.

## Glossary

AURKB: Aurora kinase B
BIRC5: Baculoviral IAP repeat-containing 5
BP: Biological processes
BUB1B: BUB1 mitotic checkpoint serine/threonine kinase B
CC: Cellular components
CCNA2: Cyclin A2
CCNB2: Cyclin B2
CDC20: Cell division cycle 20
CENPA: Centromere protein A
ChEA3: ChIP-X Enrichment Analysis 3
COVID-19: Coronavirus disease-2019
CTD: Comparative Toxicogenomics Database
DEGs: Differentially expressed genes
DLGAP5: DLG-associated protein 5
DNMT1: DNA methyltransferase 1
DPFG: Drug Perturbations from GEO
DSigDB: Drug Signatures Database
FoxO: Forkhead box, sub-group O
GEO: Gene Expression Omnibus
GO: Gene Ontology
IFN: Interferon
IL: Interleukin
JAK: STAT Janus kinase/signal transducer and activator of transcription
KEGG: Kyoto Encyclopedia of Genes and Genomes
MERS-CoV: Middle East respiratory syndrome coronavirus
MF: Molecular functions
MYBL2: MYB proto-oncogene-like 2
NSCLC: Non-small-cell lung cancer ACE2 angiotensin-converting enzyme 2
OMIM: Online Mendelian Inheritance in Man
PLK1: Polo-like kinase 1
PPI: Protein–protein interaction
RNA-seq: Transcriptomic RNA-sequencing
SARS-CoV-2: Severe acute respiratory syndrome coronavirus 2
SASP: Senescence-associated secretory phenotype
TCGA: The Cancer Genome Atlas
TFDP1: Transcription factor Dp-1
TFs: Transcription factors
TOP2A: DNA topoisomerase II alpha
TTD: Therapeutic Target Database
ZNF367: Zinc finger protein 367
