# Supplementary material for: Bioinformatics and System Biology Approach to Reveal the Interaction Network and the Therapeutic Implications for Non-Small Cell Lung Cancer Patients With COVID-19
Source: Front Pharmacol. 2022 Jun 2;13:857730. doi: 10.3389/fphar.2022.857730 (PMC9201692; doi:10.3389/fphar.2022.857730)
Supplement: Supplementary file 2 [file Table1.docx]

**Supplementary Table 1.** Antiviral and/or anticancer activities of candidate drugs for COVID-19/NSCLC from Enrichr database and ShinyGO v0.75 database.

| **Drug name** | **Virus type** | **Antiviral-mechanisms** | **Cancer type** | **Anticancer-mechanisms** | **Database** |
| --- | --- | --- | --- | --- | --- |
| Cyclosporin A | Influenza A virus | Inhibited viral assembly or budding, the function of CypA, CypB, and Pgp (Hamamoto et al., 2013). | Gastric cancer | Decreased Pgp expression and destroyed the mitochondrial outer membranes in cells (Xing et al., 2016). | DSigDB |
| Lucanthone | \ | \ | Breast cancer | Accumulated cathepsin D and enhanced cell death (Carew et al., 2011). | DSigDB and Shinny GO v0.75 |
| Troglitazone | Hepatitis B Virus | Dissociated NTCP dimers on the plasma membrane (Fukano et al., 2018). | Pancreatic cancer | Elevated caspase-3 activity, increased Bax/Bcl-2 relative expression and phosphorylation of Akt and MAPK (ERK/p38/JNK) (Fujita et al., 2017). | DSigDB |
| Coumestrol | Herpes simplex virus | Reduced the expression of  UL42 and gD proteins (Argenta et al., 2015). | Prostate Cancer | Increased phosphorylation of ERK1/2, JNK, P90RSK, and P53 proteins, induced mitochondrial dysfunction (Lim et al., 2017). | DSigDB |
| Resveratrol | MERS-CoV | Reduced the expression of nucleocapsid (N) protein and inhibited Caspase 3 cleavage (Lin et al., 2017). | Non-small cell lung cancer | Upregulated caspase-3 and decreased survivin levels (Zhao et al., 2010). | DSigDB and Drug Perturbations from GEO |
| Irinotecan | \ | \ | Colon cancer | Inhibited DNA Topo I, the activities of MDM2 and Bcl-xL (Lee et al., 2019). | DSigDB |
| Dasatinib | MERS-CoV and SARS-CoV | Inhibited kinase signaling (Dyall et al., 2014). | Chronic myeloid leukemia | Down-regulated antiapoptotic SK-1, and upregulated ceramide synthase (CerS) genes (Gencer et al., 2011). | DSigDB |
| Quercetin | Influenza A virus | Binded to HA Protein (Wu et al., 2015). | Non-small cell lung cancer | Mediated the lncRNA SNHG7/miR-34a-5p signaling pathway (Chai et al., 2021). | DSigDB |
| Genistein | Human immunodeficiency virus-1 | Modulated tyrosine kinase and TNF-a (Stantchev et al., 2007). | Ovarian cancer | Induced G2/M arrest by upregulating p21 expression (Chan et al., 2018). | DSigDB |
| Menadione | Human papillomavirus 16 | Increased ROS production, depolarization of mitochondrial membrane potential and decreased cell volume (de Carvalho Scharf Santana et al., 2016). | Colorectal cancer | Upregulated E-cadherin (CDH1), ZO-1, downregulated N-cadherin (CDH2), Vimentin (VIM), ZEB1, MMP2 and MMP9 (Kishore et al., 2019). | DSigDB |
| Trichostatin A | SARS-CoV-2 | Reduced the viral RNA load, viral antigen expression, and infectious virus particle formation (Wen et al., 2021). | Breast cancer | Decreased Cyclin D1, CDK4, CDK6 and BCL-XL, increased p21 (Song et al., 2018). | DSigDB |
| 5-Fluorouracil | Human papillomavirus 18 | Increased P53 gene and protein  expression, decreased E6 and E7 mRNA and protein expression (Didelot et al., 2003). | Colorectal cancer and breast cancer | Inhibited the nucleotide synthetic enzyme thymidylate synthase (TS) and misincorporation of fluoronucleotides into RNA and DNA (Longley et al., 2003). | DSigDB |
| Etoposide | Adenovirus | Blocked cells in the G2 phase of the cell cycle (Parsons et al., 1989). | Small-cell lung cancer | Targeted DNA topoisomerase II activities thus leading to the production of DNA breaks and dysregulated cell metabolisms (Montecucco et al., 2015). | DSigDB |
| Carboplatin | \ | \ | Non-small cell lung cancer | Promoted the formation of DNA crosslinks which interrupts cellular DNA functioning and subsequently induces apoptosis (Ho et al., 2016). | Drug Perturbations from GEO |
| Ascorbic acid | Epstein-Barr virus | Increased the production of α/β interferons and downregulated pro-inflammatory cytokines (Colunga Biancatelli et al., 2020). | Prostate cancer | Resulted in a high flux of extracellular H2O2 and initiated oxidative cascades (Zhong et al., 2004; Du et al., 2012). | Drug Perturbations from GEO |
| PD173074 | \ | \ | Hepatocellular carcinoma | Decreased P-FRS2α, P-ERK, CDK2, cyclin E and NF-κB (p65), increased ubiquitin, CUL3, and miR-141(Qiao et al., 2020). | Drug Perturbations from GEO |
| Vemurafenib | Influenza A virus | Induced a hyperactivation of the Raf/MEK/ERK cascade, p38 and JNK /MAPK pathways (Holzberg et al., 2017). | Melanoma | Binded to the ATP-binding site of BRAF V600E kinase and inhibited its activity (Garbe and Eigentler, 2018). | Drug Perturbations from GEO and ShinyGO v0.75 |
| Azacitidine | Human immunodeficiency virus-1 and Human immunodeficiency virus-2 | Blocked viral RNA or DNA synthesis (Beach et al., 2014; Rawson et al., 2016). | Myelodysplastic syndrome | Induced hypomethylation of the tumor suppressor gene cyclin-dependent kinase 4 inhibitor B (CDKN2B) (Kimura et al., 2012). | Drug Perturbations from GEO |
| Decitabine | Human immunodeficiency virus-1 | Mediated G-to-C mutagenesis (Rawson et al., 2015). | Myelodysplastic syndrome and Chronic myeloid leukemia | Incorporated into the DNA double strands in place of cytosine  and covalently traps DNMTs, resulting in the demethylation  and reactivation of methylated gene promoters (Li et al., 2015). | Drug Perturbations from GEO |
| Y15 | Influenza A virus | Inhibited FAK activity and reduced pro-inflammatory cytokines (Bergmann and Elbahesh, 2019). | Non-small cell lung cancer | Inhibited Y397 FAK autophosphorylation, activated JNK, and downregulatedBcl-2, Bcl-xL and Mcl-1 (Zhang et al., 2016). | Drug Perturbations from GEO |
| Neocarzinostatin | \ | \ | Glioma | Suppressed Akt, Bcl-2, and activated p53 (Tianqin et al., 2016). | Drug Perturbations from GEO |
| Phorbol 12-myristate 13-acetate (PMA) | \ | \ | Thyroid cancer | Modulated PKC-MAP kinase/Akt and FOXO signaling pathway (Afrasiabi et al., 2008). | Drug Perturbations from GEO |
| Palbociclib | Herpes simplex virus-1 | Regulated the pRB-E2F1-RNR2 pathway, inhibited SAMHD1 phosphorylation and blocked dNTP synthesis (Badia et al., 2016). | Breast cancer | Separated  CDK4/6-cyclin D1 complexes, blocked Rb phosphorylation,  and prevented E2F1 release (Liu et al., 2018a). | ShinyGO v0.75 |
